# Flavor diversity characterization of three loquat cultivars via flavoromics combined with E-nose and GC × GC-TOF-MS

**DOI:** 10.1016/j.fochx.2026.104235

**Published:** 2026-07-21

**Authors:** Guili Huang, Yu Shi, Xinyao Quan, Qianshuo Shao, Su Yan, Siyao Sui, Jiajia Ma, Jiayu Qian, Weiming Chai, Yuning Wang

**Affiliations:** a Institute of Agricultural Products Processing and Quality Safety, Suzhou Academy of Agricultural Sciences, Suzhou 215105, China; b Department of Science and Technology Talents, Changyinsha Modern Agriculture Demonstration Park, Zhangjiagang 215623, China; c School of Pharmacy, Jiangxi Normal University, Nanchang 330022, China

**Keywords:** Loquat cultivars, Flavoromics, E-nose, GC × GC-TOF-MS

## Abstract

Volatile Organic Compounds (VOCs) are critical determinants of loquat flavor quality and cultivar differentiation, but comprehensive characterization and rapid detection remain challenging. This study aimed to establish a robust approach for discriminating three loquat cultivars and evaluating their flavor quality by integrating electronic nose (E-nose) technology and Comprehensive Two-Dimensional Gas Chromatography–Time-of-Flight Mass Spectrometry (GC × GC-TOF-MS). GC × GC-TOF-MS was used for VOC profiling, while E-nose was applied for rapid fragrance characterization, with orthogonal partial least squares-discriminant analysis (OPLS-DA) and correlation analysis performed to identify discriminatory compounds and sensor-VOC relationships. GC × GC-TOF-MS identified over 2000 volatile compounds across three loquat cultivars. OPLS-DA further screened 22 discriminatory VOCs with variable importance in projection (VIP) values >1, which contributed to aroma hierarchy shifts among cultivars. E-nose sensors W1W and W1S showed high sensitivity to floral-scent-related VOCs, serving as key factors for distinguishing fragrance quality. Notably, E-nose detection results exhibited good consistency with GC × GC-TOF-MS data, and W1W/W1S sensors were significantly positively correlated with key VOCs. These findings demonstrate that the integration of E-nose and GC × GC-TOF-MS offers complementary advantages for comprehensive VOC characterization and cultivar discrimination. The established approach provides a scientific basis for optimizing loquat quality standards by incorporating VOC-driven biochemical authenticity and a potential efficient method for rapid, non-destructive assessment of postharvest flavor quality in loquat cultivars. These results facilitate quality control and cultivar authentication in the fruit industry.

## Highlights

• Integrate E-nose and GC × GC-TOF-MS comprehensively to characterize chemical components of three loquat cultivars.
• Discriminant VOCs form the characteristic aroma profile of the loquat cultivars.
• Core sensors were correlated with key flavor components effectively.

## 1 Introduction

The loquat (*Eriobotrya japonica* Lindl.) represents one of the most significant fruit crops in subtropical regions worldwide (Huang et al., 2022), particularly valued for its distinctive flavor profile, nutritional richness, and medicinal properties (Ayyub et al., 2025). As consumer preferences increasingly prioritize fruit quality characteristics alongside aesthetic attributes (Shen et al., 2025), understanding the complex biochemical foundations of flavor has become paramount for breeding programs and market competitiveness. While substantial efforts have been devoted to improving agronomic traits such as yield, disease resistance, and fruit quality (Zhang, Chen, et al., 2025), the flavor quality of loquats has received comparatively less scientific attention, creating a significant knowledge gap in our understanding of the metabolites and volatile compounds that contribute to their sensory characteristics.

Flavor is a complex, multidimensional fruit trait. While the measurement of fundamental indicators like sugars and acids provides a foundational assessment, traditional methods relying on them fail to capture the comprehensive volatile profile and dynamic interactions that define a cultivar's unique flavor fingerprint. These aroma compounds orchestrate the pleasant smells in food, creating distinct aroma profiles that consumers use to differentiate products. As a result, these sensory signals are a cornerstone of consumer preference, choice, and even appetite control (Chen, Sheng, et al., 2025). The flavor is advanced by complementary techniques (Cai et al., 2024). The emerging field of flavoromics addresses this limitation by employing integrated, high-throughput analytical strategies to simultaneously detect and quantify hundreds of compounds, thus defining the complete chemical landscape of food sensory properties (Iijima et al., 2024).

Electronic nose (E-nose) technology provides rapid, non-destructive pattern recognition of complex volatile mixtures, useful for distinguishing samples based on overall aroma (Shen, Cai, Ding, et al., 2023). When paired with comprehensive two-dimensional gas chromatography coupled with time-of-flight mass spectrometry (GC × GC-TOF-MS), which offers superior separation power and sensitivity, it enables a non-targeted analysis of the entire volatile profile (Bai et al., 2025). This combination effectively resolves co-eluting compounds in complex matrices and detects trace-level volatiles critical to flavor perception due to their low odor thresholds.

Recent loquat studies have begun elucidating the biochemical basis of flavor. Recent research has demonstrated that various exogenous treatments, including strigolactone (Hu et al., 2025), hydrogen sulfide (Xiang et al., 2025), cinnamon essential oil (Zhang et al., 2026), and isoleucine (Ayyub et al., 2025), effectively maintain loquat fruit flavor during storage. These treatments operate through multiple mechanisms: attenuating chilling injury by alleviating oxidative stress and maintaining membrane integrity, preventing lignification by regulating phenylpropane metabolism, and enhancing resistance to fungal infections like anthracnose. Furthermore, innovative approaches such as nanoparticle coatings with high encapsulation efficiency have shown promise in continuously suppressing flesh lignification while enhancing antioxidant capacity and cold tolerance (Jin et al., 2025). Collectively, these findings highlight the crucial roles of antioxidant system enhancement and reactive oxygen species metabolism regulation in extending loquat shelf life and preserving fruit flavor. Despite these advances, a comprehensive characterization of volatile diversity across loquat cultivars using an integrated flavoromics approach remains lacking.

To address this gap, our study employs a combined E-nose and GC × GC-TOF-MS strategy to characterize the flavor diversity of three distinct loquat cultivars. We hypothesize that each cultivar possesses a unique volatile fingerprint discernible through these techniques. Our specific objectives are to: (1) discriminate overall aroma profiles via E-nose, (2) characterize and quantify volatile compounds using GC × GC-TOF-MS, (3) identify key flavor marker compounds, and (4) correlate instrumental data with sensory attributes. The findings will provide crucial insights into the biochemical basis of loquat flavor, with applications in cultivar development and quality enhancement.

## 2 Methods and materials

### 2.1 Chemicals and reagents

Ethanol was purchased from Aladdin Biochemical Technology Co., Ltd. (Shanghai, China). n-Hexyl-d₁₃ alcohol and n-alkanes were obtained from C/D/N Isotopes INC. (Quebec, Canada) and Sigma-Aldrich (St. Louis, MO, USA), respectively. n-Hexane was supplied by Yonghua Chemical Technology Co., Ltd. (Shanghai, China).

### 2.2 Instruments and equipment

Electronic nose (E-nose): Airsense PEN3 (WinMuster Airsense Analytics Inc., Schwerin, Germany). Solid Phase Microextraction (SPME) Fiber: Divinylbenzene/carboxen/polydimethylsiloxane (DVB/CAR/PDMS)-coated fiber assembly (50/30 μm film thickness, 1 cm length), purchased from Supelco (Bellefonte, PA, USA). Gas Chromatograph (GC): Agilent 8890A (Agilent Technologies, Palo Alto, CA, USA). Mass Spectrometer (MS): LECO Pegasus BT 4D (LECO Corporation, St. Joseph, MI, USA). The GC × GC system was equipped with a LECO quad-jet dual-stage thermal modulator (LECO Corporation, St. Joseph, MI, USA), which uses liquid nitrogen for cryogenic trapping.

### 2.3 Sample preparation

Three representative loquat cultivars, Baiyu, Guanyu and Qingzhong, were selected as experimental subjects. The loquats were collected from loquat fields in Suzhou, Jiangsu Province, China. The “Baiyu” and “Guanyu” loquat cultivars were sourced from the Loquat Garden in Dongshan Town, while the “Qingzhong” variety was from the one in Jinting Town. For this experiment, loquat samples were selected following strict criteria: uniform size, free from pest infestation, disease symptoms and mechanical damage, and at a 90% ripeness level based on both fruit peel colour and total soluble solids content (TSS). After sample collection, all loquats were promptly transported to the laboratory for subsequent processing. Specifically, one experimental group was composed of 6 loquat fruits representing different cultivars, which were directly used as fresh samples for Electronic nose (E-nose) detection. The remaining loquats were cut into small segments, immediately flash-frozen in liquid nitrogen, and then stored in a −80 °C ultra-low temperature refrigerator until subsequent aroma component analysis.

### 2.4 E-nose analysis

E-nose analysis of the samples was conducted using the PEN3 E-nose system, in accordance with well-established protocols described in previous studies (Huang et al., 2023). Samples were chopped into uniform fragments via a sterile blender, and precisely 5.00 g of the fragmented sample was placed into a 20 mL crimp-top headspace bottle. Prior to E-nose determination, the samples were equilibrated at 30 °C for 30 min to allow headspace (HS) aroma accumulation. The PEN3 E-nose system is equipped with ten metal oxide semiconductor gas sensors, each exhibiting specific selectivity toward distinct classes of volatile compounds: W1C (aromatics), W5S (nitrogen oxides), W3C (ammonia and aromatics), W6S (hydrogen), W5C (alkanes and aromatics), W1S (short-chain alkanes), W1W (inorganic sulfur compounds), W2S (alcohols, ethers, aldehydes, and ketones), W2W (organic sulfur compounds), and W3S (long-chain alkanes). The E-nose analysis protocol was set as follows: cleaning time of 70 s, detection time of 70 s; clean dry air was used as the carrier gas at a constant flow rate of 400 mL/min.

### 2.5 HS-SPME-GC × GC-TOF-MS analysis

For SPME extraction, 2.0 g of fresh loquat pulp was placed into a 20 mL headspace vial and sealed with a PTFE-lined silicone septum. The sample was equilibrated at 60 °C for 10 min under agitation (250 rpm). Subsequently, a DVB/CAR/PDMS SPME fiber was exposed to the headspace and extracted at 60 °C for 30 min under continuous agitation. After extraction, the fiber was immediately inserted into the GC injector and thermally desorbed at 250 °C for 5 min in splitless mode. VOCs were analyzed using GC × GC-TOF-MS based on a modified version of a previously study (Bai et al., 2025). Analyses were performed using a LECO Pegasus® 4D instrument, which integrated an Agilent 8890A GC system (equipped with a split/splitless injector and a dual-stage cryogenic modulator) and a TOF-MS detector. A DB-Heavy Wax column (30 m × 250 μm I.D., 0.5 μm) was employed as the first-dimension column (1D), and an Rxi-5Sil MS column (2.0 m × 150 μm I.D., 0.15 μm) served as the second-dimension column (2D). High-purity helium (>99.999%) was used as the carrier gas with a constant flow rate of 1.0 mL/min. The primary oven temperature program was set as follows: initially held at 50 °C for 2 min, then ramped to 230 °C at 5 °C/min, and maintained at this temperature for 5 min. The secondary oven temperature was controlled at 5 °C higher than the primary oven, and the modulator temperature was consistently 15 °C higher than the secondary oven. The modulator was operated with a modulation period of 6.0 s. Cyclohexanone was used as the internal standard for relative quantification.

The GC injector temperature was set at 250 °C. Flavor substance analysis was conducted on the LECO Pegasus BT 4D system. The transfer line and TOF-MS ion source temperatures were both maintained at 250 °C. Mass spectral data were acquired at a frequency of 200 spectra/s. The mass spectrometer was operated in electron ionization (EI) mode with an ionization energy of 70 eV, a mass range of *m*/*z* 35–550, and a detector voltage of 1960 V. Volatile compounds were identified using the NIST 2020 mass spectral library. Following common practice in the literature, a minimum similarity of ≥90% was adopted as the acceptance threshold.

### 2.6 Statistical analysis

All experiments were conducted with at least three independent biological replicates, and the results were presented as mean ± standard deviation (SD). Principal component analysis (PCA) and bar chart construction were both performed using Origin 2021 software (OriginLab Corporation, Northampton, MA, USA). Orthogonal partial least squares-discriminant analysis (OPLS-DA) and calculation of variable importance in projection (VIP) values were carried out using Simca 14.1 (Umetrics AB, Umeå, Västerbotten, Sweden). Partial least squares (PLS) regression analysis and the Jackknife test were implemented in the R programming language using the “pls” package. Correlation network visualization was accomplished with Cytoscape 3.10.1 software.

## 3 Results and discussion

### 3.1 E-nose results analysis

**Fig. 1:**
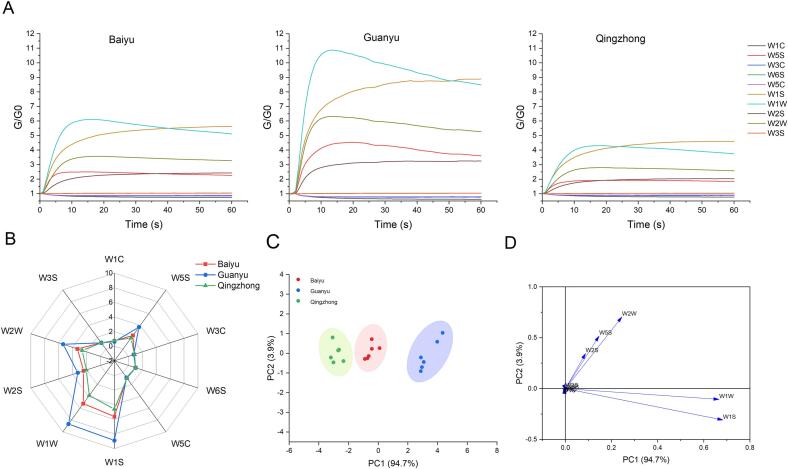
E-nose analysis of volatile profiles. (A) Response curves of the E-nose sensors over time for the “Baiyu”, “Guanyu”, and “Qingzhong” cultivars. (B) Radar plot illustrating the average response intensities of E-nose sensors across “Baiyu”, “Guanyu”, and “Qingzhong” cultivars. (C) PCA score plot of E-nose data. (D) PCA loading plot.

The E-nose enables rapid characterization of the overall volatile odor profile of samples, serving as a high-efficiency tool for volatile organic compound (VOC)-related odor assessment. Typically, each metal oxide semiconductor sensor in the E-nose exhibits higher selectivity toward a specific class of volatile compounds; the resistance ratio of each sensor is defined as the response value, which quantitatively reflects the sensor's response to target VOCs. Compared with the human olfactory system, the E-nose not only mimics its odor-sensing function but also mitigates inherent limitations of human olfaction, thereby enabling reliable recognition of both simple and complex odor matrices. In this study, the E-nose was initially employed to analyze and compare the overall odor characteristics of the three loquat cultivars (Fig. 1A). The odor radar plots of the three loquat cultivars exhibited considerable similarity, which may be attributed to two potential factors: the compositional similarity in the types of volatile components across the samples, or the insufficient sensitivity of the E-nose sensors to certain specific volatile organic compounds (VOCs). Despite this overall similarity, the total odor profiles among the three sample groups were significantly different. Among all sensors, W1W and W1S showed the highest response values, which implies relatively higher contents of inorganic sulfide compounds and short-chain alkanes in the samples, respectively. To intuitively visualize the E-nose detection results and compare the overall flavor variations among the three loquat cultivars, a radar plot was generated (Fig. 1B). Overall, the flavor profile of Guanyu loquats was characterized by enhanced sensor responses to key aroma-active compounds. Notably, the sensors within the E-nose array displayed varying response sensitivities to the volatile components of the samples, reflecting their differential detection capabilities for specific volatile compounds.

Principal component analysis (PCA) was applied to the E-nose data to identify latent patterns and discriminate sample characteristics. The first two principal components collectively explained 98.6% of the total variance, with PC1 and PC2 contributing 94.7% and 3.9%, respectively (Fig. 1C). This high cumulative variance indicates that the PCA model effectively captured the overall flavor information of the samples. The PCA score plot showed distinct separation of loquat samples according to their cultivars, confirming the E-nose's capability to distinguish between different loquat varieties. These results were consistent with the observations from the radar plots, thereby validating the robustness of the E-nose in capturing flavor differences among the samples. The loading plot from the electronic nose data for the three loquat cultivars (Fig. 1D) illustrated the contribution of each sensor to the principal components, where the distance from the origin indicated the extent of contribution-sensors farther from the origin contributed more, while those closer contributed less. Specifically, sensors W1S and W1W were relatively far from the origin, suggesting their substantial roles in differentiating the volatile profiles among the cultivars. PCA exhibited a distinct discrimination among the three loquat samples, revealing that the differences in volatile organic compounds (VOCs) across the three cultivars were statistically significant. However, a notable limitation is evident: although the E-nose technique can provide preliminary insights into the types and relative intensities of VOCs, the comprehensive characterization of detailed properties necessitates further investigation via advanced analytical methodologies.

### 3.2 Analysis of VOCs by GC × GC-TOF-MS

#### 3.2.1 Identification of VOCs

**Fig. 2:**
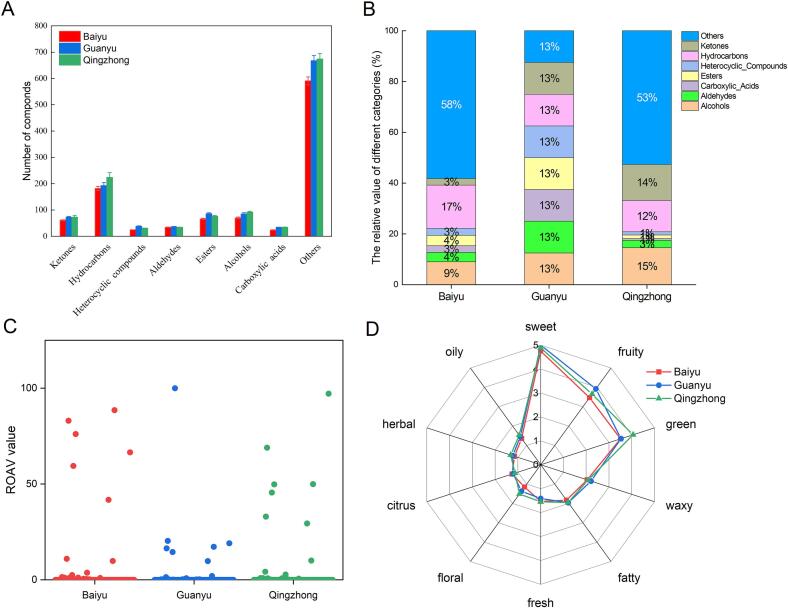
Characterization of volatile flavor profiles in three loquat cultivars. (A) Count of compounds categorized by different flavor classes. (B) Relative contents of flavor compounds in various subclasses across the three loquat cultivars. (C) Relative odor activity value (ROAV) analysis of the identified flavor compounds in the three loquat cultivars. (D) Sensory flavor characteristic analysis of the identified flavor compounds in the three loquat cultivars.

To characterize the volatile flavor compounds in three loquat cultivars, the GC × GC-TOF-MS was employed. This technique is widely recognized for its superior peak resolution (Fang et al., 2023) and enhanced accuracy in qualitative and quantitative analysis of volatile compounds in fruits (Wang et al., 2025), making it particularly suitable for this study. The identified volatile flavor compounds were categorized into eight major classes: ketones, hydrocarbons, heterocyclic compounds, aldehydes, esters, alcohols, carboxylic acids, and others (Fig. 2A). This classification is consistent with previous findings on fruit volatile profiles (Shen, Cai, Wu, et al., 2023), confirming the reliability of our detection results. However, notable quantitative variations were observed among the three cultivars across these compound classes (Fig. 2B). Specifically, Qingzhong loquats exhibited significantly higher relative contents of alcohols and ketones compared to Baiyu and Guanyu. In contrast, Baiyu loquats contained a higher relative abundance of hydrocarbons than both Guanyu and Qingzhong cultivars.

To further elucidate the contribution of VOCs to the aroma of loquats, the Relative Odor Activity Value (ROAV) of volatile flavor compounds in three loquat cultivars was evaluated in this study (Fig. 2C). Generally, a ROAV value exceeding 1 implies that the corresponding volatile compound exerts a prominent effect on the aroma. Conversely, compounds with ROAV values ranging from 0.1 to 1 are deemed to have a moderate influence on the aroma. The aroma descriptors for the volatile compounds were assigned based on the Flavornet database (http://www.flavornet.org). Furthermore, seven aroma compounds in loquats had ROAV values above 1: (E)-2-dodecenal contributed a green note, (E)-2-octenal a nutty note, heptanal a citrus note, 2,3-butanedione a fatty note, 2-pentylfuran an almond aroma, 2-methylbutanal a sweet flavor, and 1-octen-3-one a characteristic mushroom-like odor. Moreover, Baiyu and Qingzhong loquats exhibited high abundances of fruity, green, and citrus aromas, as the ROAVs of (E)-2-Octenal, Heptanal, 2-methylbutanal, 2,3-butanedione, and 2-pentylfuran were all greater than 1. Overall, Guanyu loquats demonstrated a more pronounced fruity profile compared to Baiyu and Qingzhong, while Qingzhong loquats showed an enhanced green aroma profile (Fig. 2D). These differences in ROAV value distribution among the three cultivars reflect the distinct volatile flavor compound compositions and their respective odor contributions, which is consistent with the variations observed in the types and relative contents of flavor compounds in the previous analysis (Chen, Wen, et al., 2025). However, aroma omission experiments have indicated that the OAV alone is insufficient to determine the influence of aroma compounds on the overall aroma profile. This is because the overall aroma is affected by complex interactions among compounds, including masking, antagonism, and synergy.

#### 3.2.2 Multivariate statistical analysis of VOCs

**Fig. 3:**
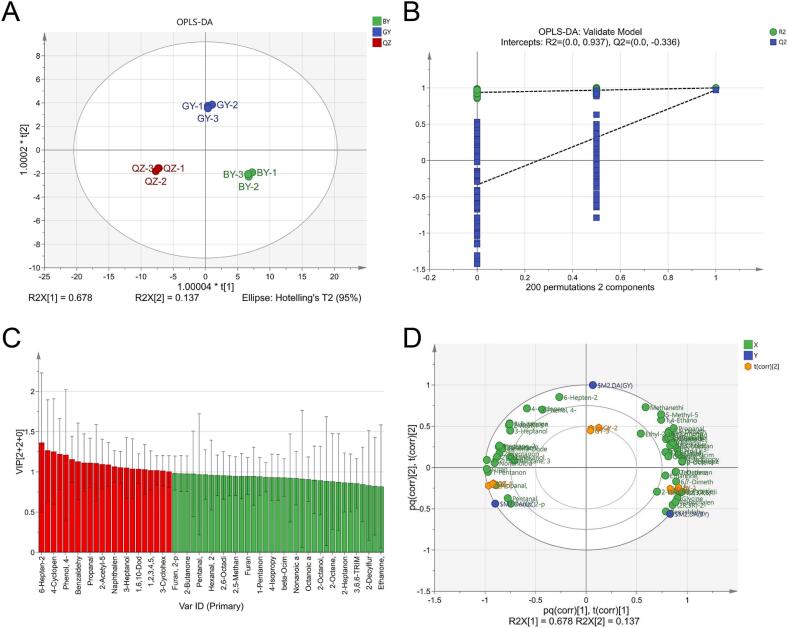
Comparative favors analysis of three loquat cultivars. (A) OPLS-DA score plot. (B) Cross-validation plot based on 200 permutation tests. (C) VIP score distribution, where red denotes characteristic flavor compounds with VIP values >1. (D) Plot of OPLS-DA loadings. (For interpretation of the references to colour in this figure legend, the reader is referred to the web version of this article.)

An orthogonal partial least squares-discriminant analysis (OPLS-DA) model was established to discriminate three loquat cultivars based on their volatile organic compound (VOC) concentrations (Zhang, Yan, et al., 2025). This model exhibited excellent fitting performance, as evidenced by its high goodness-of-fit and predictive ability parameters (R^2^Y = 0.998, Q^2^ = 0.952) (Fig. 3A). In the OPLS-DA score plot, samples of the three cultivars were distributed as relatively independent clusters, enabling effective inter-cultivar differentiation; the degree of spatial separation between clusters directly reflected the differences in VOC compositions among the cultivars. Model validity was verified via 200 permutation tests, which yielded a negative Q^2^ intercept (R^2^ = 0.936, Q^2^ = −0.336) (Fig. 3B), a critical result that ruled out the risk of model overfitting. As presented in Fig. 3B, the model's quality parameters further confirmed its good stability and robust predictive power (Zhou, Wang, et al., 2025), ensuring that the observed sample separation was driven by intrinsic cultivar differences rather than random effects. These findings further support that the three loquat cultivars can be distinguished based on their characteristic flavor profiles during the late storage stage.

Variable importance in projection (VIP) analysis was conducted using comprehensive GC × GC-TOF-MS data to identify key discriminant VOCs. A total of 22 VOCs with VIP values >1 were screened out (Fig. 3C), among which methanethiol exhibited the highest VIP score (1.40), followed by 7-phenyl-6-hepten-2-one (1.36), trans-4-cyclopentene-1,3-diol (1.25), 5-methyl-5-hexen-3-ol (1.22), and 4-ethylphenol (1.21). These compounds contributed significantly to sample classification and served as core markers for distinguishing the three loquat cultivars. To further clarify the VOCs underlying aroma differences among samples with varying storage times, a loading plot was used for in-depth analysis (Fig. 3D). It was observed that the distribution of VOCs in each sample showed distinct dispersion trends with prolonged storage; notably, 1-(2-hydroxy-3-methoxyphenyl) ethenone, furan, 1-phenyl-1-pentanone, 5-methyl-5-hexen-3-ol, propanal, and 2,6-dimethyl-2,6-octadiene emerged as key compounds driving inter-sample differentiation. Collectively, these discriminant VOCs form the characteristic aroma profile of the loquat cultivars, and the variations in their concentrations are presumably attributed to differences in metabolic accumulation or degradation rates during storage.

#### 3.2.3 Flavoromics analysis

**Fig. 4:**
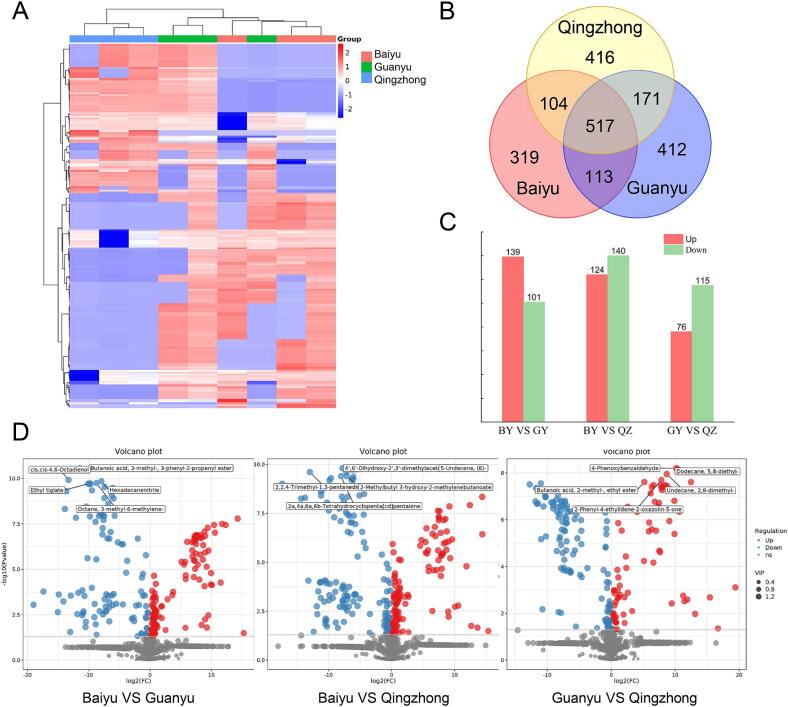
Multivariable statistics of three loquat cultivars. (A) Relative amounts of the different flavor compounds in all samples. (B) Venn diagram of flavor compounds among loquat cultivars. (C) Bar plot of flavor compounds in pairwise group comparisons. (D) Volcano plots showing differential flavor compounds.

To clarify the differences in flavor substance composition among different cultivars, this study employed a flavoromics approach to systematically analyze their volatile components. Here, GC × GC-TOF-MS analysis was conducted, which achieved clear peak separation of volatile components in the samples and ensured high stability of the detection data. To better summarize and analyze the dynamic changes in the samples' flavor substances, hierarchical cluster analysis (HCA) was further performed. As shown in Fig. 4A, the three repetitions of each cultivar were clustered together, indicating that the repeated tests of each cultivar had good homogeneity, and the detection results possessed high reliability and repeatability (Huang et al., 2024). Through database matching and CAS number annotation, a total of 2052 volatile organic compounds (VOCs) were identified, with the specific distribution presented in Fig. 4B. Among them, the “Baiyu” cultivar had 1053 VOCs in total, including 319 unique volatile components. The “Guanyu” cultivar contained 1213 VOCs, with 412 being unique to this cultivar. For the “Qingzhong” cultivar, a total of 1208 VOCs were identified, and 416 of them were unique. In addition, 517 types of volatile compounds were shared among the three cultivars. Using the criteria of VIP > 1 and *P* < 0.05, a total of 240 differential flavor compounds were identified between the “Baiyu” and “Guanyu” cultivars. Among these differential compounds, 139 were found to be up-regulated, while the remaining 101 exhibited down-regulated expression. For the comparison between “Baiyu” and “Qingzhong” cultivars, 264 differential flavor compounds were screened out under the same criteria. Specifically, 124 of these compounds showed an up-regulated trend, whereas 140 compounds displayed down-regulation. In the pairwise comparison of “Guanyu” versus “Qingzhong” cultivars, 191 flavor compounds with significant abundance differences were identified. Among them, 76 compounds were up-regulated, and 115 compounds were down-regulated (Fig. 4C and D). Among these differential compounds, benzaldehyde and propanal were the common up-regulated flavor compounds in the comparison between “Baiyu” and “Guanyu”. Meanwhile, the common three down-regulated flavor compounds in this cultivar pair were 2-butanone (PubChem CID: 6569), (*R*)-2-octanol (PubChem CID: 80080), and trans-4-cyclopentene-1,3-diol (PubChem CID: 278600). In the “Baiyu” vs. “Qingzhong” comparison, benzaldehyde (PubChem CID: 240) was identified as the common up-regulated flavor compound. The common three down-regulated flavor compounds in this pair were 2-butanone, (*R*)-2-octanol, and 2-methylpropanal. For the “Guanyu” vs. “Qingzhong” group, propanal served as the common up-regulated flavor compound. The top three down-regulated flavor compounds here were furan, 1-(2-hydroxy-3-methoxyphenyl)-ethanone (PubChem CID: 95993), and 2-methylpropanal (PubChem CID: 6561). Notably, the shared differential compounds, such as benzaldehyde (PubChem CID: 240), propanal (PubChem CID: 527), and 2-butanone, across cultivar pairs may be key contributors to the distinct flavor profiles of the three cultivars, as these compounds are known to be associated with characteristic aromas such as almond-like (benzaldehyde) and fruity (propanal) notes in fruit. Collectively, this flavoromics analysis not only confirmed the high reliability of the detection data but also systematically characterized the diversity, differential expression, and key regulatory compounds of VOCs among the three cultivars, providing a solid foundation for further elucidating the mechanisms underlying flavor formation in these varieties.

#### 3.2.4 Network interactions of VOCs and sensory aroma characteristics

**Fig. 5:**
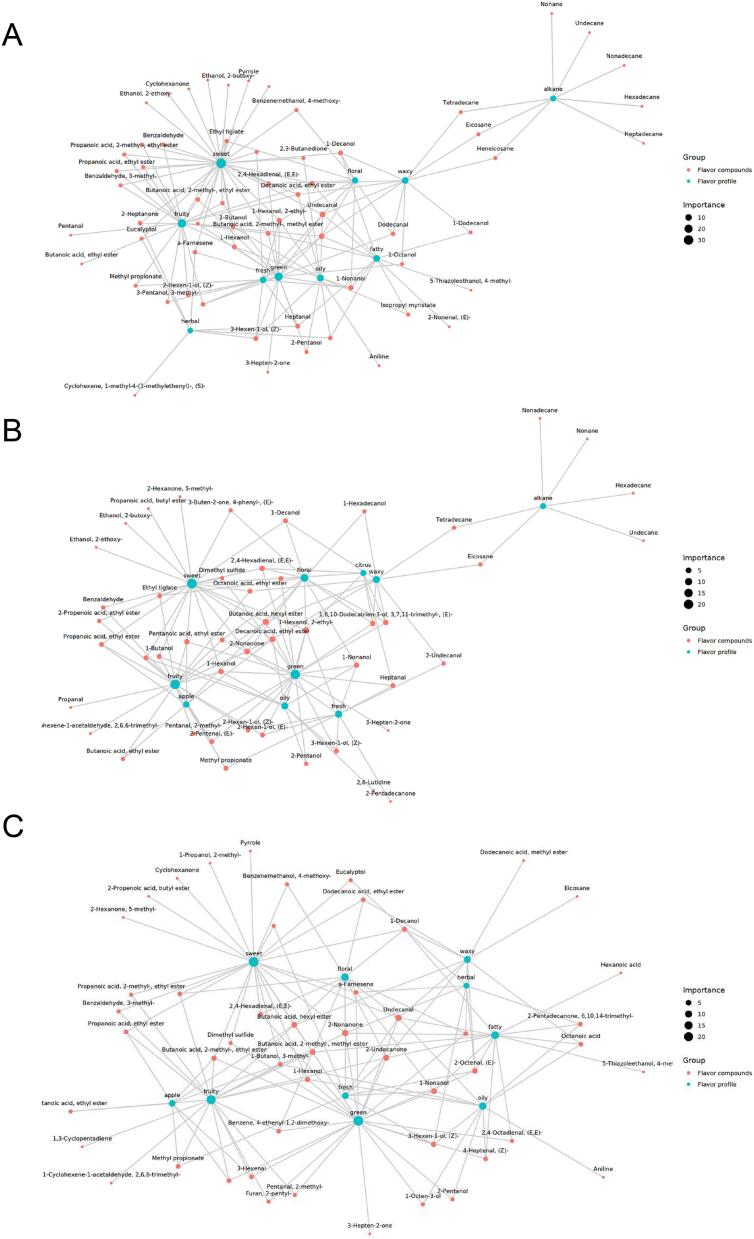
The network diagram of the association between differential VOCs and sensory flavor attributes. (A) Comparison of Baiyu and Guanyu. (B) Comparison of Baiyu and Qingzhong. (C) Comparison of Guanyu and Qingzhong. Larger blue circles denote sensory attributes connected by more VOCs; larger red circles denote VOCs contributing to more sensory attributes. (For interpretation of the references to colour in this figure legend, the reader is referred to the web version of this article.)

To systematically elucidate the complex relationship between VOCs and sensory aroma attributes in loquat cultivars, an interaction network was constructed and visualized using the Flavornet database, a comprehensive repository of flavor-active molecules and their sensory annotations (Osinomumu et al., 2025), along with the igraph software package, a powerful tool for complex network analysis and topological representation. As shown in Fig. 5, the resulting network clearly delineates the contribution patterns of significantly differential VOCs to the ten most perceptually dominant aroma characteristics of the samples. These attributes include sweet, fruity, green, waxy, floral, and fresh, among others. A key insight from the network topology is the presence of bidirectional and multi-layered connectivity between VOCs and aroma attributes. Each aroma trait is collectively influenced by a group of structurally diverse VOCs, which may act synergistically, additively, or antagonistically to shape the final sensory perception. Conversely, individual VOCs often exhibit multi-aroma specificity. For instance, hexanal, a common lipid oxidation derivative, concurrently contributes to green, fresh, and grassy notes, underscoring its role as a multifunctional aroma precursor.

This intricate interaction framework suggests that quantitative changes in the abundance of any single VOC could alter the overall aroma profile of loquat fruit, either enhancing or diminishing specific sensory notes depending on the compound's odor threshold and perceptual intensity (Zhao et al., 2025). For example, a pronounced increase in 2,3-butanedione, a key compound associated with buttery and sweet aromas, could intensify these desirable traits and thereby improve consumer acceptance. However, it is essential to recognize the inherent compensatory and neutralizing mechanisms within the VOC-aroma network. Variations in the concentration of one compound may be counterbalanced by concurrent changes in other functionally related VOCs. These mechanisms reflect the robustness of the loquat aroma profile against minor compositional fluctuations and highlight the importance of adopting a holistic, network-based perspective rather than focusing on individual compounds to accurately interpret and modulate sensory quality in loquat fruit.

### 3.3 Correlation between key volatile compounds and E-nose sensor responses

**Fig. 6:**
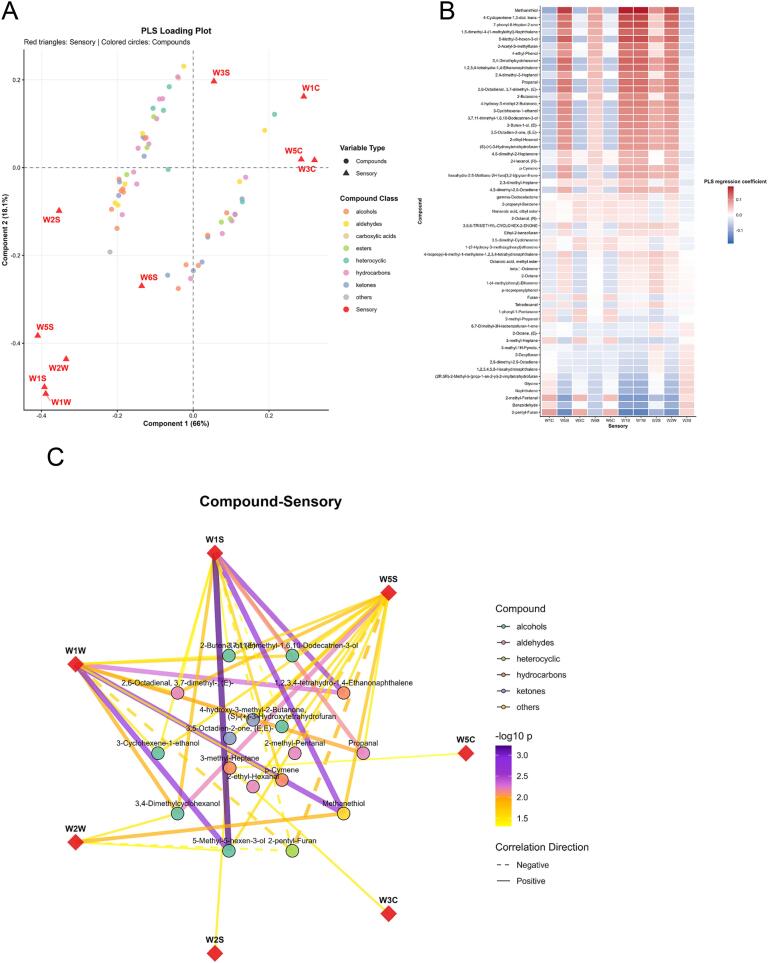
Correlation analysis between E-nose sensors and VOCs based on partial least squares regression. (A) Loading plot of PLS regression analysis. (B) HCA heatmap of PLS regression coefficients. (C) Significant correlation network between sensors and VOCs (*P* < 0.05).

To evaluate the consistency between detection outcomes from GC × GC-TOF-MS and the E-nose, partial least squares (PLS) regression analysis was performed (Zhou, Chen, et al., 2025). After preliminary screening of the detected features, 57 flavor-relevant volatile compounds were positively identified and subjected to further analysis. PLS loading plots illustrated the correlations between the 57 identified VOCs and the 10 E-nose sensors responses. Specifically, sensors W2S, W1C, and W3S exhibited strong associations with specific alcohols, hydrocarbons, and heterocyclic compounds, reflecting positive correlations (Fig. 6A). The PLS regression coefficient heatmap revealed that the majority of the 57 VOCs correlated with the 10 sensors (Fig. 6B). Specifically, W1W, W1S, W2W and W5S showed strong positive correlations with compounds such as methanethiol, trans-4-Cyclopentene-1,3-diol, 7-phenyl-6-Hepten-2-one, 1,6-dimethyl-4-(1-methylethyl)-naphthalene, 5-Methyl-5-hexen-3-ol, 2-Acetyl-5-methylfuran. A Jackknife test was performed to validate these correlations at a significance level of *P* < 0.05. This test identified 32 significant correlated pairs involving 17 distinct VOCs and 7 sensors. The significant correlation network was visualized, and the result is presented in Fig. 6C. Further classification of the 17 VOCs based on their chemical structures indicated the presence of 6 alcohols, 4 aldehydes, 1 heterocyclic compound, 3 hydrocarbons, and 2 ketones. W1S and W1W were significantly positively correlated with 5-methyl-5-hexen-3-ol, methanethiol, 1,2,3,4-tetrahydro-1,4-Ethanonaphthalene, propanal. W1W and W5S showed significant negative correlations with 2-pentylfuran. These findings not only corroborate the reliability of the PLS model but also demonstrate that the E-nose, despite its lack of compound-specific resolution, can effectively capture the overall volatile profile differences among loquat samples at different ripeness stages.

## 4 Conclusion

This study confirms that the integration of E-nose technology, GC × GC-TOF-MS, and chemometric analysis constitutes a powerful and reliable approach for discriminating three loquat cultivars based on their VOC profiles. GC × GC-TOF-MS, leveraging its enhanced separation and detection capabilities, enabled the identification of 57 vital VOCs across the cultivars, encompassing 15 hydrocarbons, 12 alcohols, 10 ketones, 7 aldehydes, 7 esters, 3 heterocyclic compounds, 1 carboxylic acid, and 1 additional VOC, providing a complete and detailed VOC fingerprint for each cultivar. Through OPLS-DA, 22 discriminatory VOCs with variable importance in projection (VIP) values >1 were identified, which are critical for distinguishing the three cultivars and explaining the aroma hierarchy shifts among them. Notably, E-nose sensors W1W and W1S exhibited remarkable sensitivity to floral-scent-related VOCs, emerging as key contributors to differentiating fragrance quality across the loquat cultivars. The detection results from the E-nose showed good consistency with those obtained via GC × GC-TOF-MS, and further correlation analysis revealed a significant positive association between W1W/W1S sensors and key discriminatory VOCs, including 5-methyl-5-hexen-3-ol and methanethiol. Furthermore, the strong correlations established between specific E-nose sensors (particularly W1W and W1S) and key volatile compound groups, such as alcohols, sulfur-containing compounds, confirm that the E-nose responses are chemically driven and can serve as reliable proxies for volatile markers. Collectively, these findings highlight the complementary strengths of E-nose and GC × GC-TOF-MS together unraveling the cultivar-specific VOC differences and aroma variations. Practically, these results provide a solid scientific basis for optimizing loquat quality standards by incorporating VOC-driven biochemical authenticity, aligning with global benchmarks for fruit evaluation. Furthermore, the established E-nose-based method presents significant potential for the efficient, rapid assessment of postharvest flavor quality in different loquat cultivars, offering valuable technical support for quality control, cultivar authentication, and market regulation in the loquat industry.

## CRediT authorship contribution statement

**Guili Huang:** Writing – review & editing, Writing – original draft, Investigation, Data curation, Conceptualization. **Yu Shi:** Resources. **Xinyao Quan:** Visualization, Validation. **Qianshuo Shao:** Visualization, Funding acquisition, Data curation. **Su Yan:** Validation, Software. **Siyao Sui:** Funding acquisition. **Jiajia Ma:** Supervision. **Jiayu Qian:** Resources. **Weiming Chai:** Supervision, Resources, Funding acquisition. **Yuning Wang:** Supervision, Resources, Funding acquisition.

## Declaration of competing interest

The authors declare that they have no known competing financial interests or personal relationships that could have appeared to influence the work reported in this paper.

## Data Availability

The data that has been used is confidential.
